# Vitamin E (α- and γ-Tocopherol) Levels in the Community: Distribution, Clinical and Biochemical Correlates, and Association with Dietary Patterns

**DOI:** 10.3390/nu10010003

**Published:** 2017-12-21

**Authors:** Sabina Waniek, Romina di Giuseppe, Tuba Esatbeyoglu, Sandra Plachta-Danielzik, Ilka Ratjen, Gunnar Jacobs, Ute Nöthlings, Manja Koch, Sabrina Schlesinger, Gerald Rimbach, Wolfgang Lieb

**Affiliations:** 1 Institute of Epidemiology, University of Kiel, 24105 Kiel, Germany; sabina.waniek@epi.uni-kiel.de (S.W.); sandra.plachta-danielzik@epi.uni-kiel.de (S.P.-D.); ilka.ratjen@epi.uni-kiel.de (I.R.); jacobs@popgen.de (G.J.); mkoch@hsph.harvard.edu (M.K.); sabrina.schlesinger@DDZ.uni-duesseldorf.de (S.S.); wolfgang.lieb@epi.uni-kiel.de (W.L.); 2 Institute of Human Nutrition and Food Science, University of Kiel, 24118 Kiel, Germany; tuba.esatbeyoglu@mri.bund.de (T.E.); rimbach@foodsci.uni-kiel.de (G.R.); 3 Biobank PopGen, University Hospital Schleswig-Holstein, Campus Kiel, 24105 Kiel, Germany; 4 Department of Nutrition and Food Sciences, University of Bonn, 53113 Bonn, Germany; noethlings@uni-bonn.de; 5 Department of Nutrition, Harvard T.H. Chan School of Public Health, Boston, MA 02115, USA; 6 Institute for Biometry and Epidemiology, Leibniz Institute for Diabetes Research, Heinrich Heine University Duesseldorf, 40225 Duesseldorf, Germany

**Keywords:** vitamin E, α-tocopherol, γ-tocopherol, dietary patterns

## Abstract

Little is known about the distribution and determinants of circulating vitamin E levels in a German population. In this cross-sectional study we assessed the distribution of both α- and γ-tocopherol levels, identified their clinical and biochemical correlates, and assessed their relationships with a priori and *a posteriori* derived dietary patterns. Plasma α- and γ-tocopherol concentrations were measured using high performance liquid chromatography (HPLC) with fluorescence detection in 641 individuals (mean-age: 61 years; 40.6% women). Correlates of both markers were determined using linear regression with backward selection. Using a validated food-frequency questionnaire (FFQ), an a priori defined vitamin E-rich dietary pattern was constructed, and three *a posteriori* derived dietary patterns were identified by principal component analysis. Each pattern was related to α- and γ-tocopherol levels using linear regression. Median concentrations of α- and γ-tocopherol were 31.54 μmol/L and 1.35 µmol/L, respectively. 57.6% of participants had α-tocopherol levels >30 µmol/L. Triglycerides, high density lipoprotein (HDL)- and low density lipoprotein (LDL)-cholesterol, and vitamin E supplementation were identified as correlates of vitamin E levels. After excluding supplement users, a dietary pattern rich in meat, bread, fats, potatoes, and sugar/confectionery was inversely related to α-tocopherol levels (β, −0.032, SE = 0.016; *p* = 0.047). Prospective studies are warranted to evaluate the actual impact of the reported findings in terms of nutrition and health outcomes.

## 1. Introduction

Vitamin E encompasses 4 tocopherols (α-, β-, γ-, and δ-tocopherol) and 4 tocotrienols (α-, β-, γ-, and δ-tocotrienol), with α-tocopherol representing over 90% of total tocopherol [1,2].

Vitamin E acts mainly as an antioxidant preventing polyunsaturated fatty acids from being damaged by lipid peroxidation [2,3]. Oxidative damage has been linked to numerous chronic diseases conditions, including cardiovascular diseases and cancer [4], and dietary vitamin E intake and lower circulating vitamin E concentrations have been linked to cardiovascular disease and cancer in observational studies [5,6,7,8,9,10,11]. However, despite the potential relevance of vitamin E for health and disease, little is known about the distribution of vitamin E levels in the general German population and about clinical and biochemical correlates of circulating α- and γ-tocopherol concentrations. To prevent nutrition-related diseases, plasma α-tocopherol concentrations of about 30 µmol/L have been recommended [12], and recommendations for the daily dietary vitamin E intake have been issued in Germany stratified by age and gender [13], but it is not well known how well these recommendations are met in the general population. In fact, a recent report from Germany indicates that about half of the analyzed population (*n* = 7532) does not meet the recommended intakes [14].

One determinant of circulating vitamin E concentrations is its dietary intake and vegetable oils, nuts, wheat germ, olives, green leafy vegetables, and fruits, which are important sources of vitamin E [2,15,16]. However, the association of vitamin E levels with dietary patterns, reflecting complex combinations of nutrients and foods that may interact in their biological effects [17], is not well explored, particularly not in Northern European populations. Only two prior US studies assessed the association between circulating plasma vitamin E and *a posteriori* derived dietary patterns [18,19].

Therefore, in the present cross-sectional study from an elderly community-based sample of Northern Germany, we aimed to assess the distribution of both α- and γ-tocopherol levels in the community, to investigate their clinical and biochemical correlates and to study the association of circulating α- and γ-tocopherol levels with both a priori- and *a posteriori* derived dietary patterns.

## 2. Methods

### 2.1. Study Sample and Design

The study sample is a subsample of the PopGen control cohort, originally encompassing 1316 participants (747 individuals from a random community based-sample and 569 blood donors) [20]. Between 2010 and 2012, a first follow-up examination was attended by 952 participants. Participants received a physical examination at the study center and provided blood samples, obtained by trained nurses. Furthermore, participants filled-in standardized questionnaires on demographics, education, smoking status, diet [21] (detailed below), physical activity (detailed below), and various health-related characteristics [20]. For the present cross-sectional analysis, we used data from this first follow-up examination.

Blood samples were taken from participants in a sitting position after overnight fasting. lithium-heparin (LH)-plasma (Sarstedt, Germany) tubes were used for triglyceride, cholesterol, and high density lipoprotein (HDL)- and low density lipoprotein (LDL)-cholesterol, plasma C-reactive protein (CRP) and glucose measurements. HbA1c was measured from potassium-ethylenediaminetetraacetic acid (EDTA) blood tubes.

For immediate laboratory analyses, unfrozen blood samples were analyzed under standard clinical conditions on the same day in the Institute of Clinical Chemistry at the University Hospital Schleswig-Holstein, Campus Kiel, and the following measurements were obtained: plasma glucose, HbA1c, total cholesterol, high HDL- and LDL-cholesterol, triglycerides, and CRP (more detailed information is provided in Supplementary Materials Table S1. Vitamin E (α- and γ-tocopherol) was measured in frozen plasma, generated from LH-blood after centrifugation (3000× *g* for 15 min, room temperature), aliquotation, and storage at −80 °C.

Plasma vitamin E concentrations were measured in a subsample of 641 study participants.

The study protocol was approved by the Ethics Committee of the Medical Faculty of the University of Kiel (Project identification code A 156/03). All participants provided written informed consent.

### 2.2. Clinical Examination and Definitions

Weight (in kg, nearest to 0.1 kg) and height (in cm, measured at one decimal) were measured with the participants wearing light clothing, using the same digital scale with height rod, between 08:00 and 12:00 in the morning. From the measured weight, 2.0 kg were subtracted to correct for the remaining clothes. Body mass index (BMI) was calculated as body weight (kg)/height (m^2^). Waist circumference (in cm, measured at one decimal) was measured at the midpoint between the lower ribs and iliac crest, and the hip circumference (in cm, measured at one decimal) was measured at the level of the trochanter major. After the participants had rested 5 min in a sitting position, blood pressure was measured twice (2 min interval) using a sphygmomanometer [22]. Average systolic and diastolic blood pressures were computed as arithmetic mean of the two measurements. Prevalent hypertension was defined as systolic blood pressure ≥140 mmHg, or diastolic blood pressure ≥90 mmHg, or use of antihypertensive medication, or self-reported hypertension history. Type 2 diabetes was defined as use of anti-diabetic medication, glycated hemoglobin (HbA1c) ≥6.5% (48 mmol/mol), or fasting serum glucose ≥126 mg/dL, or self-report physician diagnosis.

Participants also responded to validated questions [23] related to physical activity during the past 12 months including participation in several activities (walking, cycling, “do-it-yourself” activities, gardening, sports, and household chores), separately averaged for summer and winter, and the average number of stairs climbed per day. These activities were then multiplied by the corresponding metabolic equivalent of task (MET)-values and summed over all activities [24,25]. In order to assess whether participants meet the recommended dietary allowance of vitamin E, the individual dietary vitamin E intake of each person was compared to the recommended dietary vitamin E intake (stratified by age and sex), as issued by the German Nutrition Society [13].

### 2.3. Assessment of Dietary Variables

Dietary intake was assessed using a validated, self-administered, semi-quantitative 112-item food-frequency questionnaire (FFQ) designed especially for the German population [21]. Participants were asked to report the frequency of consumption of 112 food and beverage items during the previous 12 months. Nutrients and energy intakes were determined using the German Food Code and Nutrient Data Base (version II.3) and were provided by the Department of Epidemiology of the German Institute of Human Nutrition Potsdam Rehbrücke [26]. The FFQ included questions related to the use of vitamin E supplements. To reduce the arbitrariness of food-item grouping for exploratory dietary patterns analysis, food and beverage items were grouped into 39 food groups according to Kröger et al. [27].

### 2.4. Laboratory Analyses

Vitamin E (α- and γ-tocopherol) levels were determined at the Institute of Human Nutrition and Food Science from the University of Kiel in Germany using a high performance liquid chromatography (HPLC) method with fluorescence detection. Regarding the HPLC conditions, vitamin E concentrations were quantified by an external standard curve using a Jasco HPLC system (Jasco GmbH Deutschland, Gross-Umstadt, Germany; equipped with an autosampler (Jasco AS-2057; temperature 4 °C), pump (PU-2080), ternary gradient unit (LG-2080-02), 3 line degasser (DG-2080-53), and fluorescence detector (FP2020 Plus)) with a Waters Spherisorb ODS-2,3 µm column (100 × 4.6 mm) protected with a guard column. The chromatographic separation was done by isocratic elution with methanol:water (98:2, *v*/*v*) as mobile phase. The flow rate of the mobile phase was set at 1.2 mL/min and oven temperature at room temperature. The fluorescence detector operated an excitation wavelength of 290 nm and emission wavelength of 325 nm. The analytic run time was 7 min. The injection volume was set at 40 µL, and duplicate measurements of technical replications were performed. Preparing samples for analyses, plasma (50 µL) was homogenized in 2 mL 1% ascorbic acid (in ethanol), 700 µL deionised water, 50 µL 0.1% butylated hydroxytoluol (in ethanol), and 2 mL n-hexane. The samples were centrifuged (1000× *g* for 5 min at 4 °C). After separating the phases, 1000 µL of the upper phase was dried under vacuum in a RC-1010 centrifugal evaporator (Jouan, Saint-Herblain, France), and the samples were re-suspended in 200 µL mobile phase (methanol:water, 98:2, *v*/*v*) [28]. The coefficients of variation for α- and γ-tocopherol were 1.05% and 1.29%, respectively. Intra- and inter-day variations of α- and γ-tocopherol levels are provided in Supplementary Materials Table S2.

### 2.5. Statistical Analyses

Few missing values (*n* = 12) of categorical variables were replaced by the most commonly observed category of that respective variable. Missing values of normally distributed continuous variables (*n* = 10) were substituted by the respective mean, and skewed continuous variables were substituted by the sex-specific median (*n* = 2). Of the 641 CRP values, 247 were below the detection limit (0.9 mg/dL). For these values, participants were assigned a value equal to the half of the detection limit. γ-tocopherol values below the detection limit (*n* = 14) were replaced by the lowest γ-tocopherol value measured in our sample. Because vitamin E is bound to lipids in the circulation [2], the α- and γ-tocopherol/cholesterol ratios (µmol/mmol) were calculated by dividing α-tocopherol (µmol/L) and γ-tocopherol (µmol/L) concentrations by total cholesterol (mmol/L), as reported elsewhere [29].

Participants were categorized into tertiles based on the distribution of their α- and γ-tocopherol/cholesterol ratios. Mean values and the prevalence of baseline characteristics across α- and γ-tocopherol/cholesterol ratio tertiles were assessed using a general linear model, adjusting for age and sex.

### 2.6. Correlates of Circulating Vitamin E Biomarkers

Clinical, anthropometric, and biochemical correlates of plasma α- and γ-tocopherol levels and of the α-tocopherol/cholesterol ratio and the γ-tocopherol/cholesterol ratio (4 biomarkers, each considered separately) were determined using linear regression models with backward selection (variables with *p* > 0.10 were eliminated). Eligible covariates for these models were age; sex; the residual of waist circumference regressed on BMI; physical activity; systolic and diastolic blood pressure; triglycerides; CRP, HDL-, and LDL-cholesterol; HbA1c; fasting serum glucose; vitamin E supplement use; smoking status (never, current, former); education level (≤9, 10, or ≥11 years); and total energy intake. Age and sex were forced in the model, and categorical variables with more than two categories were included as indicator variables.

Correlations of vitamin E concentrations measured in plasma with the estimated intake of food groups rich in vitamin E and with estimated dietary vitamin E intake were determined using Spearman partial correlation coefficients adjusted for age, sex, and total energy intake.

### 2.7. Dietary Pattern Analyses

Two approaches were used to derive dietary patterns. First, Principal Component Analysis (PCA) was performed on 39 food groups. Briefly, PCA selects factors that explain as much predictor variation as possible [30]. The PCA works only with one set of variables, called predictors (food groups in g/day), and the number of factors that can be extracted is equal to the number of predictor variables. To identify the number of factors to retain, the Kaiser criterion (eigenvalue > 1.0) and the visual inspection of the screen plot were applied. The orthogonal varimax rotation method was used to enhance the difference between loadings, which allowed for easier interpretability.

For each participant, the extracted dietary pattern score was calculated as a sum of the products of the intake of 39 food groups with the corresponding factor loadings. Factor loadings represent the correlations of each food group with the dietary pattern score. The criterion of factor loadings was determined as greater than 0.2.

Second, an a priori dietary pattern rich in vitamin E reported by Gonzalez [31] was constructed to analyse the associations between a vitamin E-rich dietary pattern with 3 outcome variables (each outcome considered separately: (i) dietary α-tocopherol intake; (ii) circulating α-tocopherol levels (plasma); and (iii) circulating γ-tocopherol levels (plasma). Gonzalez [31] chose the top ten food groups that explained the most variance in energy-adjusted vitamin E intake to construct the food pattern rich in vitamin E.

To determine the association of *a posteriori* and a priori derived dietary patterns (exposure) with plasma α- and γ-tocopherol levels (outcome, each biomarker considered separately), a linear regression model was built. Correlations between dietary patterns (exposure) and dietary α-tocopherol intake (outcome) were tested with Spearman partial correlation coefficients adjusted for age, sex, and other covariates (see below). In addition, a sensitivity analysis was conducted by excluding vitamin E supplement users (*n* = 48). In a further sensitivity analysis, we excluded individuals with missing γ-tocopherol values (*n* = 14).

All statistical models were adjusted for total energy intake and for significant correlates of the respective biomarkers that were identified in the backward selection linear regression, as described above (“Correlates of circulating vitamin E biomarkers”). Additionally, interactions between dietary patterns and age, sex, and vitamin E supplementation were tested by including respective interaction terms into the regression models.

All analyses were conducted using SAS software version 9.4 (SAS Institute, Inc., Cary, NC, USA). All *p*-values were two-sided, and *p* < 0.05 was considered statistically significant.

## 3. Results

General characteristics of the study sample are displayed in Table 1. The median intakes of dietary α-tocopherol were 11.6 mg/day (men: 11.9 mg/day, women: 11.3 mg/day). 36.3% of men and 41.2% of women met the recommended dietary allowance for vitamin E from food as defined by the German Nutrition Society. The median levels of α-tocopherol and for the α-tocopherol/cholesterol ratio were 31.54 μmol/L and 5.53 μmol/mmol, respectively. A total of 57.6% of the study sample had adequate circulating α-tocopherol levels (above 30 µmol/L). No vitamin E deficiency (defined as <12 µmol/L) was observed in the study sample. For γ-tocopherol and the γ-tocopherol/cholesterol ratio, the median values were 1.35 μmol/L and 0.24 μmol/mmol, respectively. Overall, 7.5% of the study sample were taking vitamin E supplements.

### 3.1. Correlates of Vitamin E Biomarkers

In exploratory analyses, age- and sex-adjusted characteristics of the study sample stratified by tertiles for α- and γ-tocopherol/cholesterol ratios are shown in Supplementary Materials Tables S3 and S4. All lipid traits were strongly associated with the α-tocopherol/cholesterol ratio. Furthermore, HbA1c tended to be slightly higher, with a higher α-tocopherol/cholesterol ratio (Supplementary Materials Table S3). Similarly, all lipid traits were associated with the γ-tocopherol/cholesterol ratio. In addition, prevalent diabetes was slightly more common, whereas vitamin E supplementation was slightly less common in the top tertile of the γ-tocopherol/cholesterol ratio. Finally HbA1c, CRP, BMI, waist circumference, and triglyceride levels were slightly higher in the top compared to the bottom tertile of γ-tocopherol/cholesterol ratio levels (Supplementary Materials Table S4).

After backward elimination with age and sex forced into the models, only triglycerides, HDL-, and LDL-cholesterol and vitamin E supplementation were statistically significant correlates of α-tocopherol levels and the α-tocopherol/cholesterol ratio. The identified set of correlates explained 35.9% and 19.7% of total variation in α-tocopherol and the α-tocopherol/cholesterol ratio (Table 2). Similarly, γ-tocopherol and the γ-tocopherol/cholesterol ratio were correlated with lipid traits and vitamin E supplementation after backward elimination with age and sex forced into the models. These correlates explained 12.7% and 10.6% of total variation of γ-tocopherol and the γ-tocopherol/cholesterol ratio (Table 2).

No correlations were observed between plasma vitamin E levels and the intakes of vitamin E rich foods and dietary vitamin E intake from food. The correlation coefficient observed between plasma α- and γ-tocopherol levels and dietary α-tocopherol intake did not change in magnitude after exclusion of vitamin E supplement users (Table 3).

### 3.2. Dietary Pattern Analyses

**Figure 1:**
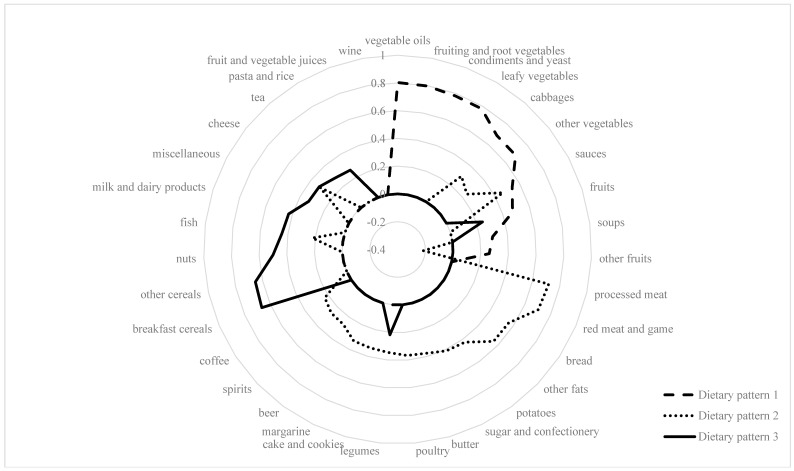
Spider web diagram from the Principal Component Analysis (factor loadings of food groups |> 0.20|).

Three major patterns were identified through PCA: dietary pattern 1 was characterized by high intakes of vegetables oils, fruiting and root vegetables, condiments and yeast, leafy vegetables, cabbages, and other vegetables. The second dietary pattern included high intakes of processed meat, red meat and game, bread, other fats, potatoes, sugar and confectionery, and butter. Dietary pattern 3 was characterized by high intakes of breakfast cereals, other cereals, nuts, fish and milk, and dairy products (Figure 1).

These patterns are explained in our sample 4.4%, 3.5%, and 2.3% variation in food intake, respectively. A full list of factor loadings from PCA-derived patterns is shown online in Supplementary Materials Table S5. In multivariable-adjusted models, both the dietary pattern 1 and the dietary pattern 3 showed a positive correlation with dietary α-tocopherol intake, which was stronger for the dietary pattern 1, whereas dietary pattern 2 showed an inverse, though weak, correlation. However, in multivariable-adjusted regression analyses, none of the PCA-derived patterns was significantly related to circulating plasma vitamin E levels (Table 4). After excluding supplement users, a borderline significant inverse association was observed between the dietary pattern 2 and plasma α-tocopherol levels (β, SE = −0.032, 0.016; *p* = 0.047). No interaction was observed between the dietary patterns and sex, age, and vitamin E supplementation in relation to plasma vitamin E levels (data not shown).

The a priori defined dietary pattern was associated with dietary α-tocopherol intake (*p* < 0.0001). However, this pattern was not related to circulating vitamin E levels in our sample (*p* = 0.475, *p* = 0.431, respectively for α- and γ-tocopherol) (Table 5).

In a sensitivity analysis, after excluding individuals with missing γ-tocopherol values (*n* = 14), the results were essentially unchanged (data not shown).

## 4. Discussion

### 4.1. Principal Findings

Our main observations were as follows: first, nearly 40% of the participants in our sample met the recommended dietary allowance for α-tocopherol from foods, as recommended by the German Nutrition Society. Close to 60% of our participants (57.6%) had adequate circulating α-tocopherol levels above 30 μmol/L. As expected, triglycerides, HDL- and LDL-cholesterol, and vitamin E supplementation were important correlates of both plasma α- and γ-tocopherol and of the ratio of each biomarker to total cholesterol. Third, we confirmed the association of a previously reported dietary pattern with dietary α-tocopherol intake. However, this pattern was not related to circulating plasma α- and γ-tocopherol levels in our sample. Similarly, three dietary patterns derived by PCA were not associated with circulating plasma α- and γ-tocopherol levels in the overall sample. One of these patterns, however, pattern 2 was inversely related to plasma α-tocopherol concentrations when supplement users were excluded.

### 4.2. In the Context of the Published Literature

#### Dietary Vitamin E Intake and Distribution of Circulating Vitamin E Levels in the Population

The dietary α-tocopherol intake in our sample (men: 11.9 mg/day, women: 11.3 mg/day) was slightly lower than in two German subgroups within the European Prospective Investigation into Cancer and Nutrition study [32]. Consistently, the proportion of individuals who met the requirement for dietary vitamin E intake (38.8%) was slightly lower than that observed in a prior study from Germany (*n* = 15,371, 52%, and 51% of men and women, respectively) [14]. In a report from the US, based on data from the National Health and Nutrition Examination Survey (NHANES), only 4.9% of men and 4.5% of women met the Recommended Daily Allowance (15 mg/day) for dietary vitamin E [33]. It has to be kept in mind that the vitamin E equivalent of the German Nutrient Database is mainly based on α-tocopherol without consideration of other vitamin E compounds. Therefore, the calculated values represent an underestimation of the actual vitamin E intake [34].

The median circulating levels of plasma α- (31.54 μmol/L) and γ-tocopherol (1.35 μmol/L) were in agreement with prior studies conducted in Germany [35,36]. Compared to data from NHANES [37], values for α-tocopherol and the α-tocopherol/cholesterol ratio in the present sample were slightly higher. Using a criterion of adequacy of 30 µmol/L [12], about 60% of individuals in our sample had adequate α-tocopherol concentrations, a proportion that is higher than in other studies in Europe (39%) and in the US (13%) [38].

Also, the levels of circulating γ-tocopherol were comparable to the results conducted in six European countries (total sample size, *n* = 2118) [39]. By contrast, most studies conducted in the US reported higher average plasma γ-tocopherol levels as compared to studies conducted in Europe [16]. This is likely explained by the fact that γ-tocopherol is the major form (≈70%) of vitamin E in the US diet [40]. The prevalence of vitamin E supplement users in our sample is slightly lower (7.5%) than in the second German National Nutritional Survey (11.4%) [14] and in a report from the US (11.4%) [41].

### 4.3. Correlates of Vitamin E Biomarkers

We identified lipid traits and the use of vitamin E supplements as key correlates of circulating α- and γ-tocopherol in our sample. The association of vitamin E levels with circulating lipid concentrations is well established and explained by the fact that vitamin E is transported in plasma in lipoproteins [2]. Also, the association of circulating vitamin E levels with the use of vitamin E supplementation is plausible. As in prior studies, α-tocopherol was positively related, and γ-tocopherol was inversely related to intake of vitamin E from dietary supplements [11,42,43].

Vitamin E consumed from supplements is predominantly in the form of α-tocopherol and may also explain increased α-tocopherol levels and decreased γ-tocopherol levels [42]. It is well documented that supplementation with α-tocopherol reduces γ-tocopherol levels in humans [44] and rodents [45]. This observation is partly explained by the function of the hepatic α-tocopherol transfer protein (α-TTP) [46], which has a higher affinity for α-tocopherol compared to other vitamin E forms (α-tocopherol = 100%, β-tocopherol = 38%, γ-tocopherol = 9%, δ-tocopherol = 2%) [47]. Once vitamin E is absorbed and taken up by the liver, the hepatic α-TTP preferentially transfers α-tocopherol into circulating lipoproteins and accounts for the higher concentration of α-tocopherol in plasma, whereas non-α-tocopherol forms are largely degraded in the liver and excreted [46,48]. An additional explanation of why γ-tocopherol levels are decreased after α-tocopherol supplementation may be found in a degradation of desmethyl vitamers via an induction of cytochrome P450 enzymes, which regulate vitamin E metabolism [45,49].

Nevertheless, in the present study the inclusion of supplement users (7.5% of the sample) in the analyses did not change the magnitude of the correlation between plasma and dietary α-tocopherol intake.

### 4.4. Lack of Association between Estimated Dietary α-Tocopherol Intake and Circulating Vitamin E Levels

One rather small study conducted in Germany [50] (*n* = 92) reported very weak correlations between plasma α-tocopherol and dietary α-tocopherol intake (*r* = 0.14), whereas others have reported higher correlations between plasma α-tocopherol levels and dietary α-tocopherol when supplement intake was taken into account [51,52,53].

In our study, however, we observed no evidence for a correlation between dietary α-tocopherol intake and circulating α-tocopherol levels, which is in line with several other studies [51,54,55]. Yet, these controversial observations may suggest that variation in circulating vitamin E levels is not only determined by dietary vitamin E intake but also by many other factors, including age, gender, lifestyle factors (e.g., smoking and alcohol consumption) [56,57], circulating lipid levels [29,46], genetic variation and variation in the absorption, metabolism, and excretion of vitamin E, as reviewed in detail elsewhere [56,57]. For example, substantial inter-individual variation has been reported regarding the intestinal absorption of vitamin E (20–80%) [56], in part explained by other dietary factors such as the intake of competing nutrients, the food matrix, or the amount of fat [15,56]. Additionally, the renal excretion of vitamin E [56], as well as its metabolism, might vary with different metabolic conditions such as obesity or the metabolic syndrome [58,59].

Further, the FFQ, the most commonly used tool to assess dietary intake in large cohort studies, does not capture all foods rich in vitamin E (e.g., olives) and reflects a very different time interval than circulating vitamin E levels. While the FFQ inquires about the food intake in the past 12 months, the half-life of α-tocopherol in plasma is 48 h, and the ingested α-tocopherol appears in plasma within 2–4 h and peaks in 5–14 h [60]. All these factors might have contributed to the poor correlation between dietary α-tocopherol intake and plasma α-tocopherol levels observed in our sample and in other studies [51,54,55], as well as to the partially conflicting results between studies [51,52,53]. Yet, we cannot completely rule out whether an unknown bias such as the unreported consumption of special or local foods might have affected the lack of correlation between vitamin E intake and plasma levels. Indeed, it will be interesting to re-evaluate the present findings once the FFQs have been recalculated when new data from the Federal Institute for Risk Assessment (BfR) Meal Study, which will cover at least 90% of the foods consumed in Germany, has been included in the newly generated FFQ database representative of the typical German eating habits.

### 4.5. Association of Dietary Patterns with Dietary Vitamin E Intake and Circulating Vitamin E Levels

We assessed the association between three dietary patterns derived by PCA and one previously reported as a vitamin E-rich pattern with dietary vitamin E intake in our sample, as well as with circulating α- and γ-tocopherol levels. The previously reported, vitamin E-rich dietary pattern, as well as dietary patterns 1–3 derived by PCA, were not associated with circulating plasma vitamin E levels in the overall sample. However, the vitamin E-rich dietary pattern and dietary pattern 1 and dietary pattern 3 displayed moderate to good positive correlations with dietary α-tocopherol intake. Given the lack of association between dietary vitamin E intake and circulating α- and γ-tocopherol concentrations (discussed above), this discrepancy in the association of the dietary patterns with dietary vitamin E intake and circulating vitamin E concentrations is not surprising.

When supplement users were excluded, circulating plasma α-tocopherol levels were only weakly inversely related to the dietary pattern (dietary pattern 2) characterized by high intakes of meat (processed meat, red meat, and game), bread, other fats, potatoes, sugar, and confectionery and butter. Only two prior US studies assessed the association between circulating plasma vitamin E and dietary patterns [18,19]. In 602 participants a “sweet” dietary pattern, which was similar to our second dietary pattern, had the lowest α-tocopherol levels relative to a “fruit and breakfast cereal” pattern and a “milk and milk products” pattern [18]. In 373 African Americans, a “juice” cluster (characterized by high intakes of fruit juice) showed higher serum α-tocopherol concentrations as compared to a “fast food” cluster [19]. In the present study, we did not identify a “juice” pattern, but fruit was part of our dietary patterns 1 and 3. Additionally, our dietary pattern 1 was characterized by high intakes of vegetable oils, the most important source of dietary vitamin E.

### 4.6. Strength and Limitations

Strengths of the present study include the population-based design, the availability of standardized information regarding dietary intake of vitamin E, and the detailed assessment of potential correlates, obtained in a standardized fashion by trained personnel. Some limitations merit consideration. Vitamin E status was defined based on α- and γ-tocopherol measurements in a single blood sample. Since dietary intake was assessed using a self-administered FFQ only, misreporting of vitamin E may have occurred. The relatively modest sample size is a further limitation.

## 5. Conclusions

In conclusion, most of the study participants in this Northern German sample had adequate plasma α-tocopherol levels, suggesting an overall adequate supply of this vitamin from the diet. However, the inverse trend observed between a “western” dietary pattern and lower plasma α-tocopherol concentrations should raise awareness that unhealthy eating patterns could negatively affect the vitamin E status. Because of the many physiological actions of vitamin E and the intense interest in possible health effects, prospective studies are warranted to confirm our results and to evaluate the actual impact of the reported findings in terms of nutrition and health outcomes.

## Figures and Tables

**Table 1:** General characteristics of the study sample (*n* = 641).

**Characteristics**	
Men, %	59.4
Age, years	61.2 (11.6)
Body mass index, kg/m^2^	27.2 (4.5)
Weight, kg	80.5 (15.7)
Hip circumference, cm	102.3 (8.8)
Waist circumference, cm	96.2 (13.0)
Systolic blood pressure, mmHg	139.6 (18.2)
Diastolic blood pressure, mmHg	85.0 (8.9)
Prevalent hypertension, %	69.0
Current smokers, %	10.9
High education (≥11 years), %	35.9
Prevalent diabetes, %	10.8
Vitamin E supplementation, %	7.5
Physical activity, MET-hour/week	90.0 (58.3, 131.6)
Alcohol consumption, g/day	9.6 (3.7, 18.7)
Dietary α-tocopherol intake (FFQ), mg/day	11.6 (9.7, 13.9)
**Biochemical features**	
α-tocopherol, μmol/L	31.5 (27.27, 37.03)
α-tocopherol > 30 μmol/L, % *	57.6
α-tocopherol/cholesterol ratio, μmol/mmol	5.53 (4.88, 6.33)
γ-tocopherol, μmol/L	1.35 (0.99, 1.79)
γ-tocopherol/cholesterol ratio, μmol/mmol	0.24 (0.18, 0.31)
HbA1c, %	5.60 (5.40, 5.90)
C-reactive protein, mg/dL	1.20 (0.45, 2.50)
HDL-cholesterol, mg/dL	65.81 (18.61)
LDL-cholesterol, mg/dL	131.36 (34.07)
Total cholesterol, mg/dL	223.42 (41.38)
Triglycerides, mg/dL	106.0 (76.0, 139.0)

MET: Metabolic equivalent; FFQ: Food frequency questionnaire; IQR: interquartile range; HDL: High density lipoprotein; LDL: Low density lipoprotein. Values are presented as mean (standard deviation), median (IQR: Q_1_, Q_3_) or percentages (%). * Participants with α-tocopherol above the criterion of α-tocopherol adequacy.

**Table 2:** Correlates for (a) α-tocopherol (b) α-tocopherol/cholesterol ratio, (c) γ-tocopherol, and (d) γ-tocopherol/cholesterol ratio.

(a) α-Tocopherol *	***β* Estimate**	**SE**	***p* Value**
Age	−0.0006	0.0007	0.358
Sex	−0.0108	0.0184	0.556
Triglycerides	0.0015	0.0001	<0.0001
HDL-cholesterol	0.0033	0.0005	<0.0001
LDL-cholesterol	0.0027	0.0002	<0.0001
Vitamin E supplementation	0.0863	0.0307	0.005
*R*^2^ = 0.359			
(b) α-Tocopherol/Cholesterol Ratio *			
Age	−0.0014	0.0007	0.040
Sex	−0.0096	0.0187	0.606
Triglycerides	0.0009	0.0001	<0.0001
HDL-cholesterol	−0.0013	0.0005	0.015
LDL-cholesterol	−0.0021	0.0002	<0.0001
Vitamin E supplementation	0.0777	0.0311	0.013
*R*^2^ = 0.197			
(c) γ-Tocopherol *			
Age	−0.0011	0.1562	0.474
Sex	−0.0681	0.0016	0.108
Triglycerides	0.0019	0.0423	<0.0001
HDL-cholesterol	0.0031	0.0003	0.011
LDL-cholesterol	0.0018	0.0012	0.001
Vitamin E supplementation	−0.3170	0.0006	<0.0001
*R*^2^ = 0.127			
(d) γ-Tocopherol/Cholesterol Ratio *			
Age	−0.0020	0.0016	0.201
Sex	−0.0454	0.0381	0.235
Triglycerides	0.0015	0.0003	<0.0001
LDL-cholesterol	−0.0030	0.0006	<0.0001
Vitamin E supplementation	−0.3225	0.0707	<0.0001
*R*^2^ = 0.106			

SE: Standard Error; HDL: High density lipoprotein; LDL: Low density lipoprotein. * Log transformed values. Variables with *p* > 0.10 were eliminated; age and sex were forced in the model.

**Table 3:** Correlations of vitamin E rich food groups and estimated vitamin E intake from food frequency questionnaire (FFQ) with plasma vitamin E adjusted for age, sex, and total energy intake.

	α-Tocopherol		γ-Tocopherol	
	rho	95% CI	rho	95% CI
Leafy vegetables	0.03	−0.05, 0.10	0.01	−0.07, 0.08
Fruiting and root vegetables	0.01	−0.06, 0.09	0.04	−0.04, 0.12
Cabbages	−0.04	−0.12, 0.04	0.05	−0.02, 0.13
Other vegetables	−0.04	−0.12, 0.04	0.03	−0.04, 0.11
Legumes	0.02	−0.06, 0.10	0.05	−0.03, 0.12
Nuts	0.08	−0.002, 0.15	0.07	−0.01, 0.15
Other fruits	0.11	0.04, 0.20	0.12	0.04, 0.19
Breakfast cereals	0.06	−0.001, 0.14	0.03	−0.05, 0.11
Other cereals	0.03	−0.04, 0.11	0.01	−0.07, 0.09
Margarine	0.01	−0.07, 0.08	0.02	−0.06, 0.10
Vegetables oils	0.06	−0.07, 0.14	0.02	−0.06, 0.10
Dietary α-tocopherol intake (FFQ)	0.01	−0.07, 0.09	0.005	−0.07, 0.08
Dietary α-tocopherol intake (FFQ) *	0.01	−0.07, 0.09	0.01	−0.07, 0.09

Values are presented as Spearman correlation coefficient with 95% Confidence Interval (CI). Other vegetables: grain and pod vegetables, onion, garlic, stalk vegetables, mushrooms, sprouts, mixed salad, and mixed vegetables; other fruits: mixed fruits, and olives; other cereals: flour, flakes, starches, semolina, dough and pastry, salty biscuits, and crackers. * Non vitamin E supplement users (*n* = 593).

**Table 4:** Multivariable-adjusted linear regression models for the association between a Principal Component Analysis (PCA)-derived dietary patterns with plasma α- and γ-tocopherol levels and dietary α-tocopherol intake.

**α-Tocopherol**									
**Overall (*n* = 641)**	**Dietary Pattern 1**			**Dietary Pattern 2**			**Dietary Pattern 3**		
	***β* Estimate**	**SE**	***p* Value**	***β* Estimate**	**SE**	***p* Value**	***β* Estimate**	**SE**	***p* Value**
α-tocopherol, µmol/L *^,†^	−0.007	0.008	0.415	−0.026	0.015	0.089	0.015	0.008	0.077
α-tocopherol/cholesterol ratio, µmol/mmol *^,†^	−0.006	0.009	0.508	−0.020	0.016	0.207	0.015	0.009	0.087
Dietary α-tocopherol intake (FFQ), mg/day ^†^	0.72 ^‡^	0.68, 0.75	<0.0001	−0.18 ^‡^	−0.25, −0.10	0.002	0.32 ^‡^	0.24, 0.39	<0.0001
**Non vitamin E supplement users (*n* = 593)**									
α-tocopherol, µmol/L *^,§^	−0.003	0.009	0.712	−0.032	0.016	0.047	0.015	0.009	0.097
α-tocopherol/cholesterol ratio, µmol/mmol *^,§^	−0.002	0.009	0.790	−0.025	0.016	0.135	0.014	0.009	0.112
Dietary α-tocopherol intake (FFQ), mg/day ^§^	0.71 ^‡^	0.67, 0.75	<0.0001	−0.16 ^‡^	−0.24, −0.08	0.0001	0.34 ^‡^	0.27, 0.41	<0.0001
**γ-Tocopherol**									
**Overall (*n* = 641)**									
γ-tocopherol, µmol/L *^,†^	0.006	0.019	0.759	−0.013	0.036	0.715	0.027	0.019	0.165
γ-tocopherol/cholesterol ratio, µmol/mmol *^,||^	0.007	0.019	0.715	−0.004	0.036	0.920	0.024	0.019	0.216
**Non vitamin E supplement users (*n* = 593)**									
γ-tocopherol, µmol/L *^,§^	0.004	0.020	0.844	0.004	0.036	0.906	0.022	0.019	0.262
γ-tocopherol/cholesterol ratio, µmol/mmol *^,¶^	0.005	0.020	0.790	0.014	0.036	0.695	0.018	0.019	0.352

SE: Standard Error; FFQ: Food frequency questionnaire. * Log transformed values. ^†^ Adjustment for: sex, age, triglycerides, HDL-cholesterol, LDL-cholesterol, vitamin E supplementation, total energy intake. ^‡^ Spearman correlation coefficient with 95% Confidence Interval. ^§^ Adjustment for all the covariates in model ^†^ excluding vitamin E supplementation. ^||^ Adjustment for: sex, age, triglycerides, LDL-cholesterol, vitamin E supplementation, total energy intake. ^¶^ Adjustment for all the covariates in model ^||^ excluding vitamin E supplementation.

**Table 5:** Multivariable-adjusted linear regression models for the association between an a prioriderived, vitamin E rich dietary pattern with plasma α- and γ-tocopherol levels and dietary α-tocopherol intake.

**α-Tocopherol**				
**Overall (*n* = 641)**	***β* Estimate**	**SE**	***p* Value**	***R*^2^**
α-tocopherol, µmol/L *^,†^	−0.002	0.003	0.475	0.359
α-tocopherol/cholesterol ratio, µmol/mmol *^,†^	−0.001	0.004	0.683	0.198
Dietary α-tocopherol intake (FFQ), mg/day ^†^	0.51 ^‡^	0.45, 0.56	<0.0001	
**Non vitamin E supplement users (*n* = 593)**				
α-tocopherol, µmol/L *^,§^	−0.001	0.004	0.749	0.355
α-tocopherol/cholesterol ratio, µmol/mmol *^,§^	−0.0002	0.004	0.947	0.179
Dietary α-tocopherol intake (FFQ), mg/day ^§^	0.49 ^‡^	0.42, 0.55	<0.0001	
**γ-Tocopherol**				
**Overall (*n* = 641)**				
γ-tocopherol, µmol/L *^,†^	0.006	0.008	0.431	0.100
γ-tocopherol/cholesterol ratio, µmol/mmol *^,||^	0.008	0.008	0.346	0.080
**Non vitamin E supplement users (*n* = 593)**				
γ-tocopherol, µmol/L *^,§^	0.005	0.008	0.549	0.107
γ-tocopherol/cholesterol ratio, µmol/mmol *^,¶^	0.007	0.008	0.412	0.084

SE: Standard Error; FFQ: Food frequency questionnaire. * Log transformed value. ^†^ Adjustment for: sex, age, triglycerides, HDL-cholesterol, LDL-cholesterol, vitamin E supplementation, total energy intake. ^‡^ Spearman correlation coefficient with 95% Confidence Interval. ^§^ Adjustment for all the covariates in model ^†^ excluding vitamin E supplementation. ^||^ Adjustment for: sex, age, triglycerides, LDL-cholesterol, vitamin E supplementation, total energy intake. ^¶^ Adjustment for all the covariates in model ^||^ excluding vitamin E supplementation.

## Supplementary Materials

The following are available online at www.mdpi.com/2072-6643/10/1/1/s1, Table S1: details about the measurements of glucose, cholesterol, high density lipoprotein (HDL)-, and low density lipoprotein (LDL)-cholesterol, triglyceride, C-reactive protein (CRP), and HbA1c; Table S2: intra- and inter-day variations of plasma α- and γ-tocopherol levels. Table S3: age- and sex-adjusted characteristics of the study sample according to tertiles (T) of α-tocopherol/cholesterol ratio; Table S4: age- and sex-adjusted characteristics of the study sample according to tertiles (T) of γ-tocopherol/cholesterol ratio; Table S5: factor loadings for food groups that highly |>0.2| loaded in principal component analysis (PCA).
