# Supplementary material for: Vitamin E (α- and γ-Tocopherol) Levels in the Community: Distribution, Clinical and Biochemical Correlates, and Association with Dietary Patterns
Source: Nutrients. 2017 Dec 21;10(1):3. doi: 10.3390/nu10010003 (PMC5793231; doi:10.3390/nu10010003)
Supplement: Supplementary file 1 [file nutrients-10-00003-s001.docx]

**Table S1: Details about the measurements of glucose, cholesterol, high density lipoprotein (HDL) - and low density lipoprotein (LDL) -cholesterol, triglyceride, C-reactive protein (CRP) and HbA1c**

| **Glucose** |  |
| --- | --- |
| TestName | Glucose HK Gen.3 (2200 Tests), Art. Nr. 05168791 190 |
| Instrument | Roche Cobas 8000/ c702 |
| Controls | PreciControl Clin. Chem. Multi 1; Roche, PreciControl Clin.Chem. Multi 2; Roche |
| Intra-assay variation | CV=max. 0.8 % |
| Inter-assay variation | CV=max. 1.3 % |
|  |  |
| **Cholesterol** |  |
| TestName | Cholesterol Gen.2 (2100 Tests), Art. Nr. 05168538 190 |
| Instrument | Roche Cobas 8000/ c702 |
| Controls | PreciControl Clin. Chem. Multi 1; Roche, PreciControl Clin. Chem. Multi 2; Roche |
| Intra-assay variation | CV=max. 0.8 % |
| Inter-assay variation | CV=max. 1.6 % |
|  |  |
| **HDL-cholesterol** |  |
| TestName | HDL-cholesterol plus 3rd generation (450 Tests), Art. Nr. 05168805 190 |
| Instrument | Roche Cobas 8000/ c702 |
| Controls | PreciControl Clin. Chem. Multi 1; Roche, PreciControl Clin. Chem. Multi 2; Roche |
| Intra-assay variation | CV=max. 0.8 % |
| Inter-assay variation | CV=max. 1.5 % |
|  |  |
| **LDL-cholesterol** |  |
| TestName | LDL-cholesterol plus 2nd generation (500 Tests), Art.Nr. 05171369 190 |
| Instrument | Roche Cobas 8000/ c702 |
| Controls | PreciControl Clin. Chem. Multi 1; Roche, PreciControl Clin. Chem. Multi 2; Roche |
| Intra-assay variation | CV=max. 0.9 % |
| Inter-assay variation | CV=max. 2.7 % |
|  |  |
| **Triglyceride** |  |
| TestName | Triglycerides (800 Tests), Art.Nr. 05171407 190 |
| Instrument | Roche Cobas 8000/ c702 |
| Controls | PreciControl Clin. Chem. Multi 1; Roche, PreciControl Clin. Chem. Multi 2; Roche |
| Intra-assay variation | CV=max. 0.9 % |
| Inter-assay variation | CV=max. 2.0 % |
|  |  |
| **CRP** |  |
| TestName | C-Reactive Protein Gen.3 (500 Tests), Art.Nr. 05172373 190 |
| Instrument | Roche Cobas 8000/ c702 |
| Controls | PreciControl Clin. Chem. Multi 1; Roche, PreciControl Clin. Chem. Multi 2; Roche |
| Intra-assay variation | CV=max. 3.7 % |
| Inter-assay variation | CV=max. 4.0 % |
|  |  |
| **HbA1c** |  |
| TestName | VARIANT™ II Turbo HbA1c Kit - 2.0, Art.Nr. 270-2455EX |
| Instrument | VARIANT™ II Turbo HPLC**-**Analyser Hemoglobin HbA1c Program, BioRad |
| Controls | Lyphocheck™ Diabetes Control, Bilevel, BioRad |
| Intra-assay variation | CV=0.78 % |
| Inter-assay variation | CV=0.53 % |
| CV: Coefficient of variation. | |

**Table S2: Intra- and inter-day variation of plasma α- and γ-tocopherol levels.**

|  | **Intra-day variation** | | | **Inter-day variation** | | |
| --- | --- | --- | --- | --- | --- | --- |
|  | **mean** | **SD** | **CV (%)** | **mean** | **SD** | **CV (%)** |
| **α-tocopherol (µmol/L)** | 34.8 | 0.264 | 0.757 | 32.4 | 2.44 | 7.53 |
| **γ-tocopherol (µmol/L)** | 1.44 | 0.056 | 3.90 | 1.33 | 0.173 | 13.0 |
| SD: Standard deviation; CV: Coefficient of variation. | | | | | | |

**Table S3: Age- and sex-adjusted characteristics of the study sample (n=641) according to tertiles (T) of α-tocopherol/cholesterol ratio .**

|  | **Tertiles α-tocopherol/cholesterol ratio** | | |  |
| --- | --- | --- | --- | --- |
| **Characteristics** | **T1** | **T2** | **T3** | **P trend** |
| n | 213 | 214 | 214 |  |
| Median α-tocopherol/cholesterol ratio (IQR), µmol/mmol | 4.63 (4.25,4.88) | 5.53 (5.36,5.72) | 6.74 (6.33,7.59) |  |
| Men, % | 54.5 | 60.7 | 61.3 | 0.181 |
| Age, years | 62.3 (60.7,63.9) | 60.6 (59.0,62.2) | 60.6 (59.0,62.1) | 0.118 |
| Body mass index, kg/m^2^ | 26.5 (25.7,27.2) | 26.7 (25.9,27.4) | 26·9 (26.2,27.7) | 0.314 |
| Waist circumference, cm |  |  |  |  |
| Men | 98.12 (95.6,100.6) | 98.4 (96.1,100.8) | 99.1 (96.7,101.5) | 0.509 |
| Women | 86.9 (83.5,90.4) | 89.2( 85.7,92.6) | 90.0 (86.8,93.2) | 0.193 |
| Hip circumference, cm | 101.3 (99.8,102.9) | 102.7 (101.2,104.2) | 102.3 (100.8,103.8) | 0.269 |
| Systolic blood pressure, mmHg | 137.4 (134.2,140.5) | 136.6 (133.6,139.7) | 136.8 (133.8,139.7) | 0.740 |
| Diastolic blood pressure, mmHg | 84.0 (82.4,85.5) | 83.0 (81.5,84.5) | 83.0(81.5,84.5) | 0.290 |
| Prevalent hypertension, % | 56.4 | 60.3 | 58.0 | 0.713 |
| Current smokers, % | 31.1 | 38.3 | 34.4 | 0.494 |
| Physical activity, MET-hour/week * | 79.2 (71.0,88.3) | 76.7 (68.9,85.3) | 85.4 (77.0,94.8) | 0.237 |
| High education (≥11 years), % | 38.8 | 47.6 | 43.0 | 0.375 |
| Alcohol consumption, g/day * | 6.5 (5.3,8.9) | 7.3 (5.9,8.9) | 8.2 (6.7,10.0) | 0.048 |
| Vitamin E supplementation, % | 4.6 | 5.9 | 9.1 | 0.097 |
| Prevalent diabetes, % | 5.2 | 5.6 | 10.8 | 0.078 |
| HbA1c, % * | 5.50 (5.41,5.58) | 5.51 (5.44,5.60) | 5.62 (5.54,5.70) | 0.014 |
| C-reactive protein, mg/dL * | 1.15 (0.96,1.38) | 1.17 (0.98,1.40) | 1.27 (1.07,1.51) | 0.365 |
| HDL-cholesterol, mg/dL | 70.93 (68.02,73.83) | 68.18 (65.33,71.03) | 62.81 (60.04,65.59) | <.0001 |
| LDL-cholesterol, mg/dL | 137.71 (132.03,143.39 ) | 126.83 (121.25,132.40) | 118.24 (112.81,123.66) | <.0001 |
| Triglycerides, mg/dL * | 88.34 (81.29,96.00) | 95.21 (87.75,103.30) | 117.47 (108.50,127.18) | <.0001 |
| γ-tocopherol, μmol/L * | 1.11 (1.02,1.20) | 1.23 (1.13,1.34) | 1.39 (1.28,1.51) | <.0001 |
| * Log-transformed variables were reported as geometric means and 95% Confidence Interval (CI). IQR: Interquartile range; MET: Metabolic equivalent; HDL: High density lipoprotein; LDL: Low density lipoprotein. | | | | |

**Table S4: Age- and sex-adjusted characteristics of the study sample according (n=641) to tertiles (T) of γ-tocopherol/cholesterol ratio .**

|  | **Tertiles γ-tocopherol/cholesterol ratio** | | |  |
| --- | --- | --- | --- | --- |
| **Characteristics** | **T1** | **T2** | **T3** | **P trend** |
| n | 213 | 214 | 214 |  |
| Median γ-tocopherol/cholesterol ratio (IQR), µmol/mmol | 0.16 (0.13,0.18) | 0.24 (0.22,0.26) | 0.35 (0.31,0.41) |  |
| Men, % | 59.7 | 59.6 | 58.0 | 0.722 |
| Age, years | 61.9 (60.2,63.4) | 60.7 (59.2,62.3) | 60.8 (59.2,62.4) | 0.389 |
| Body mass index, kg/m^2^ | 25.9 (25.2,26.7) | 26.9 (26.2,27.7) | 27.2 (26.4,28.0) | 0.004 |
| Waist circumference, cm |  | | |  |
| Men | 96.9 (94.6,99.2) | 99.0 (96.6,101.5) | 99.9 (97.5,102.2) | 0.040 |
| Women | 84.9 (81.5,88.2) | 89.2 (85.9,92.5) | 91.9 (88.8,95.1) | 0.002 |
| Hip circumference, cm | 100.6 (99.1,102.1-9 | 102.8 (101.3,104.4) | 102.9 (101.4,104.4) | 0.011 |
| Systolic blood pressure, mmHg | 136.9 (133.9,139.9) | 137.3 (134.2,140.3) | 136.5 (133.5,139.6) | 0.831 |
| Diastolic blood pressure, mmHg | 83.7 (82.2,85.2) | 83.4 (81.9,84.9) | 82.8 (81.3,84.3) | 0.323 |
| Prevalent hypertension, % | 57.1 | 62.0 | 55.7 | 0.750 |
| Current smokers, % | 35.5 | 34.6 | 33.9 | 0.726 |
| Physical activity, MET-hour/week * | 78.4 (70.5,87.1) | 82.2 (73.9,91.5) | 81.3 (73.1,90.3) | 0.561 |
| High education (≥11 years), % | 47.9 | 39.5 | 42.2 | 0.216 |
| Alcohol consumption, g/d* | 6.7 (5.5,8.2) | 7.8 (6.4,9.5) | 7.5 (6.2,9.2) | 0.354 |
| Vitamin E supplementation, % | 14.1 | 1.6 | 4.4 | 0.0002 |
| Prevalent diabetes, % | 4.2 | 6.0 | 11.9 | 0.013 |
| HbA1c, %* | 5.50 (5.42,5.59) | 5.52 (5.44,5.61) | 5.62 (5.54,5.71) | 0.014 |
| C-reactive protein, mg/dL * | 1.02 (0.85,1.21) | 1.28 (1.07,1.53) | 1.32 (1.11,1.57) | 0.012 |
| HDL-cholesterol, mg/dL | 69.56 (66.71,72.41) | 67.18 (64.29,70.06) | 64.40 (61.55,67.25) | 0.002 |
| LDL-cholesterol, mg/dL | 132.49 (126.87,138.11) | 127.26 (121.58,132.93) | 121.21 (115.61,126.83) | 0.0007 |
| Triglycerides, mg/dL * | 93.68 (86.29,101.71) | 96.79 (89.08,105.16) | 111.95 (103.13,121.52) | 0.0002 |
| α-tocopherol, μmol/L* | 30.09 (28.86,31.37) | 31.14 (29.86,32.48) | 34.10 (32.71,35.54) | <.0001 |
| * Log-transformed variables are reported as geometric means and 95% Confidence Interval (CI). IQR: Interquartile range; MET: Metabolic equivalent; HDL: High density lipoprotein; LDL: Low density lipoprotein. | | | | |

**Table S5: Factor loadings for food groups that highly |>0**.**2| loaded in principal component analysis (PCA).**

|  | Dietary pattern 1 | Dietary pattern 2 | Dietary pattern 3 |
| --- | --- | --- | --- |
| Vegetable oils | 0.80 | - | - |
| Fruiting and root vegetables | 0.80 | - | - |
| Condiments and yeast | 0.78 | - | - |
| Leafy vegetables | 0.78 | - | - |
| Cabbages | 0.69 | 0.30 | - |
| Other vegetables | 0.69 | 0.24 | - |
| Sauces | 0.54 | 0.46 | - |
| Fruits | 0.47 | - | 0.24 |
| Soups | 0.29 | - | - |
| Other fruits | 0.26 | -0.21 | - |
| Water | - | - | - |
| Processed meat | - | 0.72 | - |
| Red meat and game | - | 0.70 | - |
| Bread | - | 0.56 | - |
| Other fats | - | 0.56 | - |
| Potatoes | - | 0.43 | - |
| Sugar and confectionery | - | 0.41 | - |
| Butter | - | 0.38 | - |
| Poultry | - | 0.37 | - |
| Legumes | - | 0.35 | 0.22 |
| Cake and cookies | - | 0.34 | - |
| Margarine | - | 0.33 | - |
| Beer | - | 0.27 | - |
| Spirits | - | 0.26 | - |
| Coffee | - | 0.23 | - |
| Eggs | - | - | - |
| Other alcoholic beverages | - | - | - |
| Soft drinks | - | - | - |
| Breakfast cereals | - | - | 0.67 |
| Other cereals | - | - | 0.65 |
| Nuts | - | - | 0.50 |
| Fish | - | 0.22 | 0.44 |
| Milk and dairy products | - | - | 0.43 |
| Miscellaneous | - | - | 0.33 |
| Cheese | - | 0.31 | 0.32 |
| Tea | - | - | 0.28 |
| Pasta and rice | - | - | 0.27 |
| Fruit and vegetable juices | - | - | - |
| Wine | - | - | - |
| Only food groups with factor loadings \|>0.20\| are displayed and listed in order for simplicity and interpretation. | | | |
